# Developing an understanding of cross-protection by *Citrus tristeza virus*

**DOI:** 10.3389/fmicb.2013.00076

**Published:** 2013-04-04

**Authors:** Svetlana Y. Folimonova

**Affiliations:** Department of Plant Pathology, University of FloridaGainesville, FL, USA

**Keywords:** cross-protection, superinfection exclusion, *Citrus tristeza virus*, citrus, homologous interference

## Abstract

*Citrus tristeza virus* (CTV) causes two citrus diseases that have caused devastating losses in global citrus production. The first disease is quick decline of trees propagated on the sour orange rootstock. The second disease is stem pitting, which severely affects a number of economically important citrus varieties regardless of the rootstock used and results in reduced tree growth and vigor as well as in reduced fruit size and quality. Both diseases continue to invade new areas. While quick decline could be effectively managed by the use of resistant and/or tolerant rootstocks, the only means to protect commercial citrus against endemic stem pitting isolates of CTV has been cross-protection with mild isolates of the virus. In some citrus areas cross-protection has been successful and allowed production of certain citrus cultivars despite the presence of severe stem pitting isolates in those regions. However, many other attempts to find isolates that would provide sustained protection against aggressive isolates of the virus had failed. In general, there has been no understanding why some mild isolates were effective and others failed to protect. We have been working on the mechanism of cross-protection by CTV. Recent considerable progress has significantly advanced our understanding of how cross-protection may work in the citrus/CTV pathosystem. As we demonstrated, only isolates that belong to the same strain of the virus cross protect against each other, while isolates from different strains do not. We believe that the results of our research could now make finding protecting isolates relatively straightforward. This review discusses some of the history of CTV cross-protection along with the recent findings and our “recipe” for selection of protecting isolates.

## INTRODUCTION

*Citrus tristeza virus* (CTV) is the largest and most complex member of the family *Closteroviridae*, which contains viruses that cause severe economic losses in crops including vegetables, grains, grapes, and fruit trees (Bar-Joseph et al., 1979; Dolja et al., 1994, 2006; Agranovsky, 1996; Karasev, 2000). The natural host range of CTV is restricted to citrus and citrus relatives. Among viruses that infect citrus plants, CTV has been the most destructive. Following the large dissemination from its origin, which is thought to be South East Asia, into new regions at the end of nineteenth century due to active movement of different citrus varieties between continents, the virus caused severe disease epidemics in citrus and nearly destroyed whole citrus industries in several countries around the globe (reviewed by Moreno et al., 2008). Furthermore, in many citrus growing regions severe isolates of the virus continue to limit citrus production.

As the only option to suppress some of the aggressive virus isolates after they become endemic, cross-protection with mild isolates has been extensively explored in different production areas (reviewed by da Graça, 2010; Roistacher et al., 2010). Earlier attempts to use this approach had erratic results. When successful, the mild protecting isolates have enabled the commercial production of certain citrus varieties in some citrus areas. However, protecting isolates have not been found in other regions or for other varieties. In many cases mild CTV isolates failed to protect or provided only short-term protection against severe disease.

Elucidation of the mechanism of CTV cross-protection has been an important component of the research program in our laboratory for a number of years. In this review I discuss some of the history of CTV cross-protection that goes back more than half of a century along with the recent findings of our research.

## THE COMPLEX OF CTV DISEASES

Depending on the virus isolate and a citrus host scion/rootstock combination, CTV causes two major diseases, which have had a major impact on global citrus production. The first disease is quick decline of trees on the sour orange (*Citrus aurantium* L.) rootstock, which results from a virus-induced graft incompatibility between the scion and rootstock. During the last century severe epidemics of CTV-caused quick decline that developed in citrus growing regions destroyed almost 100 million trees (reviewed by Moreno et al., 2008). These losses prevented further usage of this popular rootstock for propagation of trees in citrus areas where decline-causing isolates of CTV were endemic. Alternative rootstocks, which create scion/rootstock combinations that do not respond with the decline syndrome to such virus isolates, were put in use. Although this allowed effective management of CTV-induced quick decline, those rootstocks often did not perform as well as the well-adapted sour orange rootstock.

Another disease that is caused by some of the CTV isolates is stem pitting. The disease severely affects grapefruit (*C. paradisi* Macfadyen), sweet orange [*C. sinensis* (L.) Osbeck], and lime [*C. aurantifolia* (Christm.) Swingle] trees regardless of the rootstock used. Stem pitting results from disrupted differentiation of the cambium as the stem of an infected tree grows, which leads to the development of pits in areas of virus multiplication (Brlansky et al., 2002; Tatineni and Dawson, 2012) resulting in reduced tree growth and vigor as well as in reduced fruit size and quality, which are highly important economic concerns (Roistacher and Moreno, 1991; Garnsey et al., 2005; Moreno et al., 2008). The CTV-associated stem pitting has caused significant economic damage for citrus industries in many different countries, including Brazil and other countries in South and Central Americas, South Africa, Australia, and a number of countries in Asia. In most of these regions stem pitting remains to be a major factor limiting citrus productivity.

Both diseases continue to spread into new areas, mainly via movement of infected plants or vegetative propagation of infected budwood followed by further local spread by several aphid species (Hilf et al., 2007; Moreno et al., 2008; Matos et al., 2013). There have been multiple examples of inadvertent introduction of severe CTV into many citrus-producing countries due to the international movement of citrus varieties despite established quarantine practices (Moreno et al., 2008). The discovery of new exotic CTV isolates in commercial citrus plantings in California (M. Polek and R. Yokomi, personal communication) and in Florida, USA (Sieburth and Nolan, 2005; Hilf et al., 2007) represent some of the recent examples. Once introduced, new isolates can be readily dispersed within a region via natural transmission of the virus by its aphid vector. The potential for future crop losses from CTV is much greater than what has been seen to date. Therefore, the development of means to protect citrus plantings against aggressive isolates is critical for virus suppression.

## MANAGING CTV DISEASES VIA CROSS-PROTECTION

Cross-protection, a phenomenon in which a pre-existing viral infection prevents a secondary infection with the same or closely related virus, was first demonstrated by McKinney (1926, 1929) between two genotypes of *Tobacco mosaic virus*. Since then, cross-protection has been observed often for viruses of different taxonomic groups, including bacteriophages and animal viruses, for which the phenomenon was commonly referred to as homologous interference or superinfection exclusion (Salaman, 1933; Bennett, 1951; Dulbecco, 1952; Visconti, 1953; Steck and Rubin, 1966a, b; Bratt and Rubin, 1968; Hull and Plaskitt, 1970; Fulton, 1978; Adams and Brown, 1985; Delwart and Panganiban, 1989; Lecoq et al., 1991; Wen et al., 1991; Strauss and Strauss, 1994; Karpf et al., 1997; Singh et al., 1997; reviewed by Hull, 2002; Lee et al., 2005; Gal-On and Shiboleth, 2006). With plant viruses, cross-protection was initially used as a test of virus relatedness to define whether two virus isolates were “strains” of the same virus or represented different viruses (McKinney, 1929; Salaman, 1933; reviewed by Hull, 2002; Gal-On and Shiboleth, 2006). Subsequently purposeful infection with a mild isolate was implemented as a protective measure against endemic isolates of the virus that caused severe disease, which in some cases was called “pre-immunization” (reviewed by Hull, 2002; Gal-On and Shiboleth, 2006). The practical aspect of the cross-protection phenomenon is reflected in the more focused definition of the phenomenon used by Gonsalves and Garnsey (1989) as well as a number of other researchers, who described cross-protection as “the use of a mild virus isolate to protect plants against economic damage caused by infection with a severe challenge strain(s) of the same virus.” The ability of mild isolates to protect against challenge with other isolates of the same virus has been demonstrated for a large number of plants viruses (reviewed by Ziebell and Carr, 2010). However, practical measures for virus suppression in the field were developed for only a few of them. In addition to CTV, some of the examples of viruses for which such applications were shown to be successful include *Zucchini yellow mosaic virus *in squash, melon, and watermelon (Cho et al., 1992; Yarden et al., 2000), *Cacao swollen shoot virus* in cocoa (Hughes and Ollennu, 1994), *Tomato mosaic virus *in tomato and pepper (Tien and Zhang, 1983), and *Papaya ringspot virus* in papaya (Yeh et al., 1988). In most cases, however, the use of cross-protection was eventually abandoned due to the breakdown of protection or development of alternative control means, such as generation of resistant plants. Remarkably, one of the first examples of the commercial exploitation for prevention of severe viral infections was cross-protection against severe CTV stem pitting with mild virus isolates (Grant and Costa, 1951). Cross-protection has continually played a major role in maintaining profitability of citrus production in several industries around the world (reviewed by Moreno et al., 2008).

Among the two diseases caused by CTV, stem pitting is the most difficult to control. The disease affects both scion and rootstock, so changing to tolerant rootstocks is not effective. At present, the only means to protect commercial citrus varieties from severe CTV-associated stem pitting is cross-protection with appropriate mild CTV isolates. This approach has been most extensively used in Brazil where more than 80 million Pera sweet orange trees are protected. It also has been used in Australia for protection of Marsh grapefruit against severe stem pitting isolates widely distributed in the country as well as for protection of Star Ruby grapefruit in South Africa, Navel orange and lime in Peru, red grapefruit in Argentina, and *C. hassaku* trees in Japan where it allowed commercial production of those citrus varieties despite the presence of aggressive stem pitting isolates in those regions (reviewed by da Graça, 2010; Roistacher et al., 2010).

With all the successes in the use of cross-protection described above, an enormous difficulty of making cross-protection work needs to be understood. The reality is that without knowing rules of CTV cross-protection it was very hard and in most cases impossible to find protecting isolates. In Brazil, for instance, it took over a decade and half for the establishment of commercial orchards of cross-protected Pera sweet orange (Costa and Müller, 1980). Prior to finding a satisfactory mild isolate, many sweet orange, lime, and grapefruit plantations were surveyed in order to identify trees that were doing well in groves severely affected by the stem pitting disease. Forty five selections were used for further field tests that involved almost 2,300 trees. Among those 45 mild isolates, only six were satisfactory, which included three for Pera sweet orange, two for Galego lime, and one for Ruby Red grapefruit. Results, however, varied depending on the source of the isolate as well as the variety of the plants tested. Thus, mild isolates from Pera sweet orange did not provide protection in lime or grapefruit trees. Similarly, the best isolates for Pera sweet orange were collected from trees of the same cultivar (Costa and Müller, 1980). Furthermore, similar mild isolate protection approaches had minimal or no success in other regions or with other varieties. In South Africa a search for protecting isolates to preserve profitability of the Star Ruby grapefruit industry was initiated in the late 1970s and is still continuing (van Vuuren and Manicom, 2005; Roistacher et al., 2010). Mild isolates that were initially selected for the interim protection proved unsuitable in field trials over several years. Host specificity of cross-protection efficiency was also noticed, even to a much greater extent. Most mild isolates derived from grapefruit cultivars other than Star Ruby performed poorly in this cultivar, with one exception of an isolate collected from a Redblush grapefruit tree. The latter isolate is the present pre-immunizing isolate for grapefruit in South Africa. Mixed results were obtained in Australia. Trials using a few mild isolates were conducted over a period of 20 years in two distinct field sites. In some cases the degree of protection appeared to be affected by climate, with breakdown in cross-protection being less in the hotter inland site than on the coast (Broadbent et al., 1991). Although an acceptable degree of Marsh grapefruit protection was achieved, difficulties have been experienced in pre-immunizing red grapefruits and no mild isolates that could confer protection against stem pitting of sweet orange were found (Broadbent et al., 1991; Zhou et al., 2002). Complete lack of success in developing cross-protection-based means to control CTV was reported in California. There it proved highly difficult to find local mild isolates of the virus that would protect against severe stem pitting isolates. Evaluation of over 100 mild isolates collected from throughout California yielded no protection (Roistacher and Dodds, 1993).

In addition to the efforts to develop effective protection against stem pitting, extensive experimentation has been done in order to achieve protection against quick decline. As discussed above, in contrast to the stem pitting disease, quick decline could be effectively managed by the use of resistant and/or tolerant rootstocks in combination with pathogen-free germplasm. This, however, does not negate an importance of finding mild virus isolates that could provide sustained protection against this disease. Due to the high adaptability of sour orange rootstock to a variety of soil types and its tolerance to the oomycetes-associated root rot diseases as well as the ability to support scions that produce high yields of fruit, it would be desirable in many situations to preferentially use this rootstock. The development of an effective cross-protection strategy against quick decline would bring it back into play. A number of experiments were conducted in this attempt worldwide, however, all were unsuccessful, and no effective protective CTV isolate has been found (reviewed by da Graça, 2010; Roistacher et al., 2010).

Overall, finding protecting isolates has been empirical and rarely successful. The general approach for selecting protecting isolates was to find infected plants showing little or no symptoms in areas where severe isolates have caused serious disease and test them for the ability to protect against severe isolates in different varieties, which required years of evaluation. Researchers have spent their whole careers trying to develop a cross-protection-based approach to control CTV. Often mild CTV isolates failed to protect or provided only limited short-term protection against severe disease. Best results were obtained when mild isolates derived from certain citrus varieties were used for pre-immunization of the same varieties; the same isolates usually performed poorly when were used with other citrus varieties. In general, there has been no understanding why some mild isolates were effective and others failed to protect.

## UNDERSTANDING CROSS-PROTECTION BY CTV

### EXAMINATION OF THE ABILITY OF DIFFERENT ISOLATES OF CTV TO PREVENT SUPERINFECTION BY ANOTHER ISOLATE OF THE VIRUS

**FIGURE 1:**
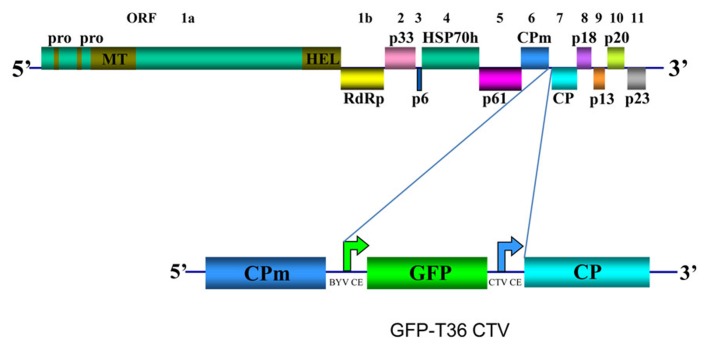
**Schematic diagram of the genome organization of wild type CTV and its derivative GFP-T36 CTV encoding green fluorescent protein (GFP).** The open boxes represent ORFs and their translation products. PRO, papain-like protease domain; MT, methyltransferase; HEL, helicase; RdRp, an RNA-dependent RNA polymerase; HSP70h, HSP70 homolog; CPm, minor coat protein; CP, major coat protein. Bent arrows indicate positions of *Beet yellows virus* (BYV) or CTV CP sgRNA controller elements (CE).

CTV has long flexuous virions (2000 nm × 10–12 nm) that are encapsidated by two coat proteins. A single-stranded RNA genome of CTV, which is ~19.3 kb, encodes twelve open reading frames (ORFs; Pappu et al., 1994; Karasev et al., 1995) (**Figure 1**). ORFs 1a and 1b are expressed from the genomic RNA and encode polyproteins required for virus replication. ORF 1a encodes a 349 kDa polyprotein that has two papain-like protease domains plus methyltransferase-like and helicase-like domains. Translation of the polyprotein is thought to occasionally continue through the polymerase-like domain (ORF 1b) by a +1 frameshift. Ten 3′ end ORFs are expressed by 3′ co-terminal subgenomic RNAs (sgRNAs; Hilf et al., 1995; Karasev et al., 1997). Those ORFs encode the following proteins: major (CP) and minor (CPm) coat proteins, p65 [heat shock protein 70 (HSP70) homolog], and p61 that are involved in assembly of virions (Satyanarayana et al., 2000); a hydrophobic p6 protein with a proposed role in virus movement (Dolja et al., 2006; Tatineni et al., 2008); p20 and p23, which along with CP are suppressors of RNA silencing (Lu et al., 2004); and p33, p13, and p18, which play a role in extending the virus host range (Tatineni et al., 2011). Yet, trees of most citrus varieties can be infected with mutants that have the genes for the latter three proteins deleted (Tatineni et al., 2008).

CTV has numerous isolates with distinctive biological and genetic characteristics. The isolates can be classified into six major CTV genotype groups or strains: T3, T30, T36, VT, T68, and resistance breaking (RB), with some isolates being unclassified (Folimonova et al., 2010; Harper, this series). Strains are defined as phylogenetically distinct lineages of CTV based upon analysis of nucleotide sequences of the 1a ORF (Hilf et al., 2005; Folimonova et al., 2010; Harper, this series). This region of the genome displays high genetic diversity between CTV variants, with levels of sequence identity ranging between 72.3 and 90.3% (Mawassi et al., 1996; López et al., 1998; Kong et al., 2000; Rubio et al., 2001; Hilf et al., 2005; Roy et al., 2005; Harper et al., 2010). This compares to a range of 89–94.8% identity found in more conserved 3′ half regions of the genomes of isolates from different CTV strains. Each strain is composed of isolates with minor sequence divergence, generally less than 5% throughout the entire genome (Hilf et al., 2005; Moreno et al., 2008; Harper et al., 2010). Isolates of a strain, however, may have significant variations in symptoms and symptom severity. Remarkably, field trees usually contain complex populations of CTV, which are often composed of mixtures of different genotypes and recombinants between these genotypes (Grant and Higgins, 1957; López et al., 1998; Kong et al., 2000; Rubio et al., 2001; Vives et al., 2005; Weng et al., 2007; Martïn et al., 2009).

Earlier we developed a green fluorescent protein (GFP)-expressing CTV vector based on an infectious cDNA clone of CTV T36, the type isolate of the T36 strain (Folimonov et al., 2007; GFP-T36 CTV herein). This virus contains an extra ORF, that of GFP inserted into the viral genome between the CPm and CP ORFs under the control of the CP sgRNA controller element (CE) from *Beet yellows virus* (**Figure 1**). The biological characteristics of GFP-T36 CTV in citrus trees were nearly identical to that of the wild type T36. Both viruses showed similar time intervals for developing systemic infections and produced similar symptoms in infected plants (Folimonov et al., 2007). Multiplication of GFP-T36 CTV in different citrus varieties produced GFP fluorescence, observation of which allowed visualization of virus distribution in phloem-associated cells of those hosts (Folimonova et al., 2008). The engineered GFP-tagged T36 CTV has been used as a tool in examination of the relationships between different isolates of CTV in terms of cross-protection.

The definition of cross-protection has evolved over time. It was first used to describe the phenomenon of the inability of a second virus to establish infection in a host that is already infected with another isolate of the same virus. Cross-protection also has been viewed as amelioration of symptoms of a severe virus isolate by pre-inoculation of a host with a mild isolate. We define cross-protection as superinfection exclusion or, in other words, as the ability of a primary virus infection to completely exclude secondary infection with the same or closely related virus.

**FIGURE 2:**
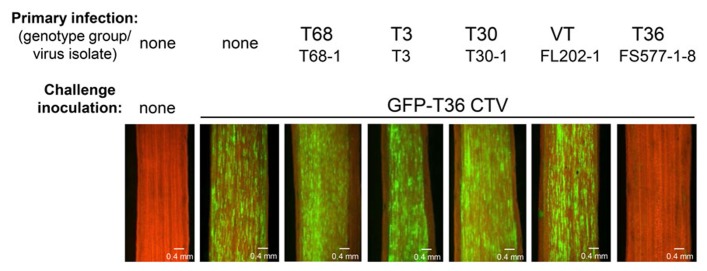
**Observation of GFP fluorescence in phloem-associated cells of *C. macrophylla* trees upon challenge with GFP-T36 CTV.** Left panel represents a non-inoculated healthy tree. Other panels represent trees with no primary infection (second panel) or pre-infected with isolates belonging to five CTV strains, which were sequentially challenged with GFP-T36 CTV. Observations were done on the internal surface of bark at 2 months after challenge inoculation using a dissecting fluorescence microscope. Scale bar = 0.4 mm. **Figure 2** as it appears in this review is similar to that published in the original manuscript (see Figure 3 in Folimonova et al., 2010).

After examination of many different CTV isolates, it was found that superinfection exclusion occurs between isolates of the same strain, but not between isolates of different CTV strains (Folimonova et al., 2010). When citrus trees pre-infected with an isolate of one of the five genotypes (strains) of CTV (T30, T3, T68, VT, or T36) were sequentially challenged with GFP-marked T36 CTV, all of them with the exception of the plants that were initially infected with isolates of the latter T36 genotype displayed GFP fluorescence similar to that observed in control plants that had no primary infection and were inoculated only with the challenge virus (**Figure 2**). The isolates of heterologous strains had no interference with the secondary infection by the T36-based virus. In contrast, no GFP fluorescence was detected in plants first infected with isolates of the T36 strain. The T36 isolates completely prevented superinfection by the GFP-tagged virus of the same T36 strain. The results were “black and white.” The isolates from heterologous strains conferred no protection. The isolates from the same strain protected totally. Additional experiments in which interactions of several different combinations of primary and challenging virus isolates were evaluated using reverse transcription polymerase chain reaction (PCR)- or serology-based differentiation between genotypes of the virus demonstrated that CTV isolates that have established a systemic infection in citrus trees prevent superinfection by an isolate of the same strain, but not by isolates from different strains (Folimonova et al., 2010). Remarkably, similar results were obtained using two different citrus hosts for CTV: highly susceptible *C. macrophylla* and less susceptible sweet orange in which fewer cells become infected with the virus compared with the former host. In both hosts exclusion among isolates of the same strain of CTV was absolute, while isolates from different strains demonstrated complete lack of exclusion. Furthermore, with the GFP-marked virus used as a challenge virus, we saw no difference in the proportion of cells infected or in the intensity of GFP fluorescence per infected cell in trees infected initially with isolates of heterologous strains compared to inoculation of trees with no primary infection. The isolates of heterologous strains that were established initially appeared to have no effect on infection, movement, and replication of the challenge virus. Additionally, when trees were initially infected and later challenged with isolates belonging to the same strain, there was no evidence of infection and replication of the challenge isolate in any of the trees.

As discussed above, isolates of CTV are generally classified into phylogenetically distinct lineages or strains based on sequence analysis of the more diverged 5′ half of the genome (Harper, this series). This grouping reflects the pattern of exclusion, suggesting the sequence divergence in this region of the genome may affect inter-virus interactions resulting in the complete lack of superinfection exclusion between isolates of different CTV strains. This contradicts with the premise of one of the original uses for superinfection exclusion as a measure of virus relatedness, in which non-excluded viruses were identified as different viruses (Matthews, 1991). Apparently, that is not the case with CTV. Superinfection exclusion defines excluding CTV isolates as members of the same strain, not different strains.

### EXCLUSION OF SUPERINFECTION BY ISOLATES OF CTV IN THE FIELD

The findings from our basic research discussed above correlate well with other observations that we have made while analyzing the dynamics of CTV populations in the Dominican Republic. Our data demonstrated a dramatic change in CTV populations that occurred in this region over a period of 10 years, which was characterized by tremendous increase in the incidence of the VT genotype and the introduction of two new virus genotypes, T36 and RB (Matos et al., 2013). Remarkably, the VT isolates of CTV were able to move in and spread in commercial citrus despite the fact that prior to their introduction into the country most citrus trees have been already infected with mild T30 isolates of the virus. The pre-existing isolates of the T30 genotype apparently did not provide protection against the isolates of the VT genotype. The same was true for the newly found T36 and RB genotypes. These viruses appeared to be able to superinfect trees that appeared to be infected with other genotypes of the virus prior to their invasion. Multiple infections of trees resulted in formation of complex virus populations composed of various combinations of different genotypes. Since a systemic infection with a CTV isolate in citrus trees prevents superinfection by an isolate of the same genotype, but not by isolates from other genotype groups of the virus, the widely spread isolates of the T30 genotype could not prevent dissemination of the isolates of the VT and T3 genotypes that were introduced in the Dominican Republic later. Further, the pre-existing infection with isolates of all these genotypes could not exclude invasion of isolates of the two other genotypes, the T36 and RB.

### POTENTIAL MECHANISMS

Superinfection exclusion of viruses has been related to a number of different mechanisms acting at various stages of the viral life cycle, including prevention of the incoming virus entry into cells (Steck and Rubin, 1966a, b; Lee et al., 2005), competition between primary and challenging viruses for host factors and intracellular replication sites, interference with disassembly, translation or replication of the secondary virus (Steck and Rubin, 1966a, b; Sherwood and Fulton, 1982; Adams and Brown, 1985; Abel et al., 1986; Karpf et al., 1997; Lu et al., 1998; Beachy, 1999; Lee et al., 2005), and induction of RNA silencing by the protector virus that leads to sequence-specific degradation of the challenge virus RNA (Ratcliff et al., 1997, 1999; reviewed in Hull, 2002). Most of the proposed mechanisms, with the exception of the latter one, could function only in cells that were infected with the primary virus, leaving uninfected cells susceptible to the secondary virus. Based on our data, such mechanisms would not be relevant for superinfection exclusion by CTV, since the phenomenon appears to be systemic and functions not only in cells infected with the primary virus, but also in cells that were not infected. Usually, in a host, CTV infects only a portion of the phloem-associated cells: less than one-third of the cells even in the most susceptible varieties (Folimonova et al., 2008). However, even though the majority of cells were not infected by the primary isolate, exclusion of a challenging isolate of the same strain was absolute. Not only the one-third of the cells that contained the primary virus was protected, but the other two-thirds of the cells that were not infected became “immune” to the challenging virus (Folimonova et al., 2010). Thus, the exclusion phenomenon must be able to spread beyond the infected cells.

The “systemic” nature of superinfection exclusion by CTV parallels characteristics of RNA silencing that has been considered as the major antiviral defense mechanism in plants and invertebrates (Vance and Vaucheret, 2001; Voinnet, 2001, 2005; Baulcombe, 2004; Li and Ding, 2005). RNA silencing can be triggered systemically: in cells that contain the primary virus and also in cells that were not pre-infected with the one. The mechanism elicits degradation of RNA molecules that have nearly identical sequences (Ratcliff et al., 1999; Jan et al., 2000; Thomas et al., 2001; Voinnet, 2001). Therefore, for a number of plant viruses RNA silencing was suggested as a mechanism that confers homologous interference of viruses (Ratcliff et al., 1997, 1999; Valkonen et al., 2002; reviewed by Hull, 2002; Gal-On and Shiboleth, 2006).

To examine the role of RNA silencing in CTV superinfection exclusion, we attempted to trigger exclusion between heterologous CTV isolates by substituting extended regions in the genome of the protecting virus with the exact cognate sequences from the genome of the challenging virus. The substituted regions contained 3′ end genes, which amplify large amounts of double-stranded RNAs (Moreno et al., 1990, 2008; Hilf et al., 1995). This part of CTV genome directs production of most viral small RNAs upon CTV infection (Ruiz-Ruiz et al., 2011). Nevertheless, the hybrids in which these regions were substituted from the challenge isolate failed to exclude the latter isolate despite that they shared extended identical sequences (Folimonova et al., 2010). These results did not appear to support the RNA silencing-based model and further argued for the intriguing complexity of CTV superinfection exclusion phenomenon, posing a possibility of an existence of a novel mechanism for superinfection exclusion between virus variants.

Most recently, we demonstrated that superinfection exclusion by CTV is due to a mechanism that requires production of a specific viral protein, the p33 protein (Folimonova, 2012). The p33 is a non-conserved protein with no significant homology to other known proteins and is not essential for CTV infection in most citrus hosts (Tatineni et al., 2008). Lack of the functional p33 completely abolished the exclusion ability of the virus. The virus mutants that failed to produce p33 failed to exclude superinfection by the parental wild type virus. Superinfection exclusion was conferred by the protein rather than the RNA sequence: the mutants that retained the entire sequence of the p33 ORF, yet, had a deletion of the subgenomic mRNA CE for the p33 sgRNA or a frameshift mutation within the p33 ORF failed to exclude the wild type virus. The plants pre-infected with the p33 mutants and sequentially challenged with the GFP-marked CTV showed GFP fluorescence, which distribution and intensity were comparable to that found upon inoculation of trees with no primary infection (Folimonova, 2012). More studies will be needed to determine whether superinfection exclusion by CTV involves components of RNA silencing pathway or operates via another novel mechanism.

The p33 protein appears to function in a homology-dependent manner. The hybrid viruses with the p33 substitutions behaved, similarly, to the mutants that produced no p33. They were unable to interfere with the secondary infection by the wild type virus, indicating that a heterologous p33 could not confer the exclusion (Folimonova, 2012). These data suggest an existence of a precise interaction(s) of the p33 protein with some other viral factor(s) involved in superinfection exclusion.

## RECIPE FOR CROSS-PROTECTION BY CTV

As a result of our research efforts, now we know the basic rule of CTV cross-protection: sustained protection against a severe isolate of a particular CTV genotype (strain) can be achieved only by using mild isolates of the same genotype. We believe that this knowledge could make finding protecting isolates relatively straightforward. The first objective for development an effective cross-protection system is to identify the genotype of the severe isolate that needs to be controlled. Then a mild isolate of that same genotype needs to be found. If such an isolate does not occur naturally, it is possible through recombinant DNA methodologies to map the disease determinant(s) of the severe isolate and then remove it by substituting sequences from a mild isolate of a different strain. The resulting mild isolate should exclude the severe isolate. A similar approach was used for the decline isolate in Florida, USA (Albiach-Martï et al., 2010).

To fulfill the first objective, or, in other words, to identify the “enemy,” an assessment of the pathogenic potential of CTV isolates in a given area needs to be conducted. This includes collection of CTV isolates from highly symptomatic trees in various locations and their biological characterization using standard indicator hosts (grapefruit, sweet orange, sour orange, and Mexican lime) and commercially important varieties. The following step is molecular characterization of those isolates in order to determine their genotype composition. At first, this can be done by amplifying genomic fragments with the oligonucleotide primers that specifically amplify sequences of particular CTV genotypes (strains) using nucleic acids extracted from collected plant material, followed by sequence analysis of the resulting products. We have used a similar strategy for characterization of CTV populations in the Dominican Republic (Matos et al., 2013). The approach has been also widely used by many other CTV researchers (Rubio et al., 2001; Hilf et al., 2005; Roy and Brlansky, 2009; Scott et al., 2012). An alternative strategy, which recently became quite popular among different virologists, is the use of next-generation sequencing techniques for virus characterization (Wu et al., 2010; reviewed by Singh et al., 2012). Sequencing of full viral genomes could be done, for instance, via using viral small RNAs that are produced during infection. Those are purified and used for library construction, which is then subjected to a high-throughput sequencing that generates millions of short reads in a single sequencing run. The latter reads are further used for virus genome reconstruction via methods of computational analysis. This approach was recently used for analysis of CTV isolates from Spain and Florida, USA (Ruiz-Ruiz et al., 2011; Harper, this series). Similarly, viral genome sequencing via next-generation sequencing techniques could be conducted using cDNA prepared from total or double-stranded RNA isolated from virus-infected plants as has been demonstrated in a number of recent publications (Adams et al., 2009; Coetzee et al., 2010).

For the second objective, non-symptomatic trees in which CTV is detected will be of particular interest, since such trees may contain desirable mild CTV isolates. The genotype composition of those isolates could be characterized using the same approaches as described above. The basic rule for selection of a protecting isolate is that the mild isolate has to have a similar genotype composition as the severe one that needs to be controlled. If a severe isolate contains a mixture of several different genotypes, then a mild isolate that contains a similar genotype mixture needs to be found. Additionally, knowledge needs to be obtained about what genotype in the severe isolate is responsible for disease symptoms. For this purpose, attempts to separate individual genotypes by single aphid transmission or passaging through selective citrus hosts should be conducted, followed by biological characterization of the resulting isolates using indicator hosts coupled with their molecular characterization. Once it becomes known which genotype causes the disease, a mild isolate containing the same genotype could be put in use to trigger exclusion of the former variant.

## FUTURE CONSIDERATIONS

Overall, our data demonstrate that superinfection exclusion by CTV is an active virus-controlled function. It is a powerful process that completely prevents a challenging infection by a closely related virus variant. At this point, its effectiveness is limited to isolates belonging to the same virus strain. However, because severe isolates of the virus frequently represent a mixture of different virus strains, for practical applications to control CTV diseases in the field, it would be valuable to develop a broad-spectrum cross-protection, for instance, by creating a virus for protection against multiple CTV strains. Our premise is that further research on the superinfection exclusion mechanism will define ways for more effective protection of citrus crop against CTV, including engineering transgenic resistance and developing methods to extend the effectiveness of cross-protection. Knowledge developed with CTV can be further transferred to other viruses that cause diseases in other economically important crops.

## Conflict of Interest Statement

The author declares that the research was conducted in the absence of any commercial or financial relationships that could be construed as a potential conflict of interest.
